# Brazil’s Community Health Workers Practicing Narrative Medicine: Patients’ Perspectives

**DOI:** 10.1007/s11606-021-06730-8

**Published:** 2021-04-07

**Authors:** Rogério Meireles Pinto, Rahbel Rahman, Margareth Santos Zanchetta, W. Galhego-Garcia

**Affiliations:** 1 School of Social Work, University of Michigan, Ann Arbor, MI USA; 2 Graduate School of Social Service, Fordham University, New York, NY USA; 3 Daphne Cockwell School of Nursing, Ryerson University, Toronto, Canada; 4 Department of Basic Sciences, Faculty of Dentistry of Araçatuba, Estadual Paulista University, São Paulo, Brazil

**Keywords:** community health workers, Brazil, narrative medicine, Unified Health System

## Abstract

**Background:**

Narrative medicine (NM) encourages health care providers to draw on their personal experiences to establish therapeutic alliances with patients of prevention and care services. NM medicine practiced by nurses and physicians has been well documented, yet there is little understanding of how community health workers (CHWs) apply NM concepts in their day-to-day practices from patient perspectives.

**Objective:**

To document how CHWs apply specific NM concepts in Brazil’s Family Health Strategy (FHS), the key component of Brazil’s Unified Health System.

**Design:**

We used a semi-structured interview, grounded in Charon’s (2001) framework, including four types of NM relationships: provider–patient, provider–colleague, provider–society, and provider–self. A hybrid approach of thematic analysis was used to analyze data from 27 patients.

**Key Results:**

Sample: 18 females; 13 White, 12 “Pardo” (mixed races), 12 Black. We found: (1) provider–patient relationship—CHWs offered health education through compassion, empathy, trustworthiness, patience, attentiveness, jargon-free communication, and altruism; (2) provider–colleague relationship—CHWs lacked credibility as perceived by physicians, impacting their effectiveness negatively; (3) provider–society relationship—CHWs mobilized patients civically and politically to advocate for and address emerging health care and prevention needs; (4) provider–self relationship—patients identified possible low self-esteem among CHWs and a need to engage in self-care practices to abate exhaustion from intense labor and lack of resources.

**Conclusion:**

This study adds to patient perspectives on how CHWs apply NM concepts to build and sustain four types of relationships. Findings suggest the need to improve provider–colleague relationships by ongoing training to foster cooperation among FHS team members. More generous organizational supports (wellness initiatives and supervision) may facilitate the provider–self relationship. Public education on CHWs’ roles is needed to enhance the professional and societal credibility of their roles and responsibilities. Future research should investigate how CHWs’ personality traits may influence their ability to apply NM.

## INTRODUCTION

While medical providers (“providers”) have technological and scientific knowledge, they have been described as lacking humility and empathy in their interactions with patients of care and prevention services. This shortcoming has resulted in decreased health care engagement and medical adherence,^1^ lower health care satisfaction,^2^ and adverse health outcomes, namely, poor HIV-related and psychosocial outcomes,^3, 4^ lack of cancer screenings,^5^ among others. Charon (2001) suggested that providers ought to engage in humane and effective narrative medicine (NM),^6^ a practice in which providers treat their patients in a fashion that allows them to uncover psychosocial issues corresponding to patients’ illnesses and to recognize, absorb, interpret, and act based on the narratives (stories) told by their patients about their illnesses.^6^ To achieve the goals of NM, providers are encouraged to listen genuinely to patients’ stories and those shared by patient’s friends, family, caretakers, and other members of the health care team, and also to observe and inquire about the patients’ facial expressions and body language.^7^ By engaging with patients’ narratives about their illnesses, providers can better understand their life circumstances and the impact of the environment on their health.^8^

Narrative medicine has evolved from medical humanities (history, philosophy, ethics, literature, literary theory, the arts, and cultural studies), primary care and patient-centered care, biopsychosocial medicine, holistic care, and psychoanalysis.^9^ There is a general consensus in the literature, often focused on physicians and nurses, that medical providers can best serve patients when they have the capacity to self-care; be empathetic, compassionate, and trustworthy; probe narratives and discern patients’ facial and verbal expressions; and have insight on the socio-political influences on patients’ health outcomes.^10^ The literature suggests that NM has the potential to help providers improve in all those areas.^11, 12^

Missing from the literature is evidence on how community-based providers (such as community health workers (CHWs)) apply key concepts of NM.^13^ Most research on NM concerns theoretical articles or critical reviews; they are limited to American viewpoints on health sciences.^14^ The literature on CHWs relies on self-reported studies on their perceptions and attitudes, with a more current understanding of providers’ and administrators’ perceptions of how best to integrate CHWs into health care teams.^15, 16^ This paper focuses on patients’ experiences of engaging with CHWs in order to provide insights into the relationships between providers with patients, with other providers, with society in general, and also with themselves.^17^ We used Charon’s NM framework^18^ to explore Brazil’s Unified Health Systems patients’ perspectives on how CHWs apply NM concepts in four types of personal and interpersonal relationships, detailed below.

### Conceptual Framework

In the absence of a NM framework for CHWs, we base this research on Charon’s existing model suggesting the use of textual and/or narrative data to understand better how a medical provider can engender satisfying relationships with patients, colleagues, society, and themselves.^18^ We also base this research on previous work findings with CHWs in Brazil serving patients with whom they share similar life stories.^19^ Therefore, we use Charon’s framework, from the perspectives of patients, to explore how CHWs, who are members of Brazil’s Unified Health System health teams (along with nurses and physicians), and listen and respond to the complexities of patients’ struggles. This framework allowed us to seek patients’ understanding of how CHWs can alleviate human suffering and offer humane care by valuing professional relationships with patients, colleagues, society, and themselves.

#### Provider–Patient

In NM, providers reflect on and relate to patients’ lived experiences beyond the proximal reasons that may bring them to care.^6^ This practice requires that health care providers develop the ability to engage in several NM concepts—altruism, compassion, respectfulness, humility, courage, trust, empathy, attentiveness, and patience.^6, 10^ Providers are concerned with both clinical (e.g., physical evaluation and study of laboratory reports) and patients’ narratives of illness.^6^ This process can produce a more precise diagnosis, trust in health care providers, and acceptance of medical advice.^10^ By creating a space for patients to express ideas, concerns, expectations, feelings, and emotions, a patient is more likely to tell the whole story of their illness and to ask questions about it.^6^ Good rapport between patients and providers helps providers understand more holistically patient behavior and how sociocultural and political environments may contribute to patients’ medical conditions.

#### Provider–Colleague

Narrative medicine calls for collaborative relationships between providers within an organization and across health care systems. To achieve better outcomes for patients, different providers must rely on one another for their respective expertise. It is recommended that health care providers be mutually respectful as they engage in decision-making concerning the optimal care of shared patients.^7^ To cultivate collegial relationships, providers should be aware of colleagues’ identities (e.g., gender, sexuality, age, work experience), leadership styles, norms, roles, and decision-making process.^7^ A recognition of unequal power between providers (e.g., physicians and nurses) and addressing it may enhance providers’ awareness of their actions as they embrace humility, generosity, kindness, and thoughtfulness with providers of other care practices and status.^18^ Doing so can allow for providers to be open towards receiving feedback on their competency and ethical judgments and then work to improve on these fronts.^7^

#### Provider–Society

Narrative medicine encourages providers to learn about social determinants of health (SDOH) impacting their patients’ health.^18^ Health care disparities are rooted in well-established SDOH—income inequality, poor quality or overcrowded housing, food insecurity, and inadequate access to preventive care,^20^ as well as environmental issues like lack of garbage disposal facilities and poor sewerage systems.^21^ This ecological understanding calls for a holistic approach to care and prevention, including learning about historically underserved populations mistreated by a racist health care system,^21^ and advocating for community activism for social and environmental changes.^7^

#### Provider–Self

Narrative medicine encourages health care providers to monitor their own motives and behaviors influencing their treatment of patients, including being aware of one’s own vulnerabilities (e.g., low self-esteem, power, and status differentials). This self-awareness can help providers address preconceived notions and prejudices they may have about patients.^7^ Focusing on personal illness and vulnerability promotes a more empathic understanding of the totality of patients’ health.^22^

### Community Health Workers in Brazil’s Unified Health System

The framework above suggests that the behaviors and interactions between different types of health providers and patients can be influenced by the sociocultural and political environments around them. This study focuses on community health workers (CHWs) in Brazil. Therefore, we provide an overview of CHWs and the health care system in which they operate.

Community health workers are frontline public health workers who are often members of the community they serve, and they thus share similar life experiences and similar socioeconomic and medical needs.^23, 24^ In middle-income countries, the majority of medical (triage) and social services have been task-shifted to CHWs.^25^ The CHWs serve as a liaison between individuals, communities, and health and social services providers; they conduct home visits lasting long periods of time; they are involved with patients’ day-to-day care; and they engage in community activism aimed at socio-political changes.^26^

CHWs’ effectiveness has been well documented, such as reducing health disparities,^27^ maternal mortality,^28, 29^ rates of HIV/AIDS,^30, 31^ tuberculosis,^32^ and malaria.^33^ CHWs’ influence has been shown to increase uptake of immunizations^34^ and cancer screenings^35^ and they contribute to the management of asthma,^36^ diabetes,^37^ and cardiovascular illnesses.^38^ CHWs’ effectiveness is attributed partly to their knowledge of the communities they serve, their use of colloquial language, and their enhanced understanding of patients’ life experiences, in addition to their capacity to engender trust and empathy.^39^

In Brazil, CHWs are referred to as Agentes Comunitários da Saúde (Community Health Agents); however, for ease of comprehension, we will use the term CHWs. There are 265,000 CHWs working across 43,000 Family Health Teams serving 209 million people in Brazil.^40^ CHWs were introduced in 1989 in response to the constitutional mandate that health is a human right and the government ought to provide health care to all.^41^ CHWs were formally integrated into the public health system in the 1990s through the Family Health Program (FHP)—the central strategy used by Brazil’s Unified Health System to provide basic prevention and primary care.^42^ In 2002, CHWs were recognized as a profession.^42^

Funded by the federal government, the FHP delivers health services by a multidisciplinary team in community-based health units across the country. The team comprises a minimum of one physician, one nurse, one auxiliary nurse, and four to six CHWs. Each team provides primary care to approximately 3500 to 4500 residents in specified geographic areas.^43^ Each CHW is responsible for 750 individuals and is expected to visit each household at least once a month.^43^ CHWs are responsible for enrolling families in the FHP; updating demographic and health information on patients; identifying individuals exposed to health risk factors; motivating patients to attend health-related appointments; explaining laboratory test results to patients; motivating patients to adhere to medication regimens; scheduling appointments with health providers; collecting data for the governmental health database; and administering various disease prevention psychoeducation groups.^44^ Other roles include social mobilization and community development.^43^ These responsibilities reflect those described for CHWs across the globe.^45–48^

## METHODS

### Sampling and Recruitment

This study was approved by the appropriate IRBs in Brazil and the USA. The Family Health Program (FHP), in the town where this research took place, includes nine community-based care units, all of which participated in the study. Our study had a purposive sample with an average of three patients per unit. The nurse administrator screened patients to ensure that they met the following inclusion criteria: enrolled and received services by FHP CHWs in the past 12 months; between 21 and 75 years of age; able to read Portuguese and understand the informed consent; and currently not being treated for mental illnesses. Using a list of potential research patients, a trained research assistant randomly contacted patients, explained the nature of the study, and invited them to participate. We sampled for maximum diversity to include a balance of male and female patients and different age groups. Brazil’s research policy does not allow for a monetary incentive. As per qualitative research norms, the authors decided to stop sampling and end data collection when they determined the study reached saturation, which means that no new insights emerged from the data.^49^

### Data Collection

Interviews were conducted by six trained, Portuguese-speaking Brazilian interviewers, holding at least a BA degree and had received training in research methods. Interviews were conducted in Portuguese in private spaces. Interviews lasted 60–75 min, were digitally recorded and transcribed by Portuguese-speaking transcriptionists, and were entered into Nvivo 10 for data management and coding. The transcriptions were translated from Portuguese to English and iteratively back-translated into Portuguese.^50^

We used a semi-structured interview guide, including one question about the types of services CHWs provided to residents, followed by open-ended questions according to the contours of Charon’s framework describing NM key personal and interpersonal relationships: provider–patient, provider–colleague, provider–society, and provider–self.^18^ Protocol questions included “How has the informal and personal approach of CHWs helped change your health habits and adapt healthier habits?”; “Do you understand CHWs as members of the FHS team?”; “How do you feel CHWs contribute to society’s well-being?”; and “Can you please describe how you view CHWs and their own well-being?”

### Analytic Approach and Data Interpretation

A hybrid approach of thematic analysis was used as it incorporated the deductive a priori template of codes based on the four NM relationships and then a data-driven inductive approach.^51^ We made this decision based on our previous research with CHWs whose data suggested that the way CHWs engage with patients is very similar to goals attributed to NM, namely to listen to, engage with, and act based on patients’ stories about their illnesses (see Pinto et al. for details).

A deductive approach was used to analyze the data, involving the building of a preliminary code book based on the four primary relationships. Transcripts were independently coded by the co-authors. We met to discuss the framing of the initial findings in relation to the four NM relationships and recognized that sub-themes emerged from the data. We then used inductive coding to develop specific codes reflecting key NM concepts—trust, humility and compassion, empathy, patience and respect, attentiveness, altruism, interprofessional collaboration (IPC), community mobilization, and CHW well-being—expected to be present within the NM relationships in question. Through co-coding and consensus, we arrived at 100% agreement after an iterative and reflexive process.^52^ Quotes used below were selected independently, compared for accuracy, and edited for clarity by the first author, who is fluent in both English and native Portuguese.

## RESULTS

### Demographic Characteristics

Table 1 shows sample demographics. Most patients identified as female (*n*=18; 67%), and married (*n*=19; 70%). Patients identified as White (*n*=13; 45%), Pardo (*n*= 12; 44%), and as Black (*n*=2; 7%). Ages ranged from 21 to 75 years, with 23 patients older than 50 years. Seventy-seven percent of patients (*n*=21) had been served by the FHS between 1 and 12 years.

**Table 1:** Demographic Characteristics and History of Care (*N*=27)

	Total (%)
Gender	
Female	18 (67)
Male	9 (33)
Race	
Black	2 (7)
White	13 (48)
Pardo	12 (45)
Age	
21–30	1 (4)
31–40	3 (11)
41–50	11 (41)
51–75	12 (44)
Education	
Some elementary school	9 (33)
Elementary school	6 (22)
High school	9 (33)
College	3 (12)
Employment status	
Employed	6 (22)
Unemployed	2 (7)
Retired	4 (15)
Self-employed	5 (19)
Homemaker	7 (26)
Retired	3 (11)
Marital status	
Single	3 (11)
Married	19 (70)
In a relationship	3 (11)
Divorced/separated	2 (8)
Length of time enrolled in FHS (years)	
1–12	21 (77)
13–24	5 (19)
Number of CHWs who provided care	
1–2	16 (60)
3–4	6 (22)
5 or more	5 (18)
Years under CHW care	
1–5	14 (53)
6–10	11 (40)
More than 10	2 (7)

### CHWs’ Practice of Narrative Medicine

Patients described CHWs’ roles as bringing prescribed medications to their home during house visits; advising them on how to keep their homes clean to prevent diseases such as dengue; inquiring about other family members; assisting with diabetes management; and offering psychoeducation about health activities and public health information. Patients underscored CHWs’ abilities to make linkages to other health care services, such as specialty doctors, laboratory tests, and to mobilize the community to create systemic changes. Below, we provide descriptions and examples of *how* CHWs may practice NM key concepts with the four types of relationships described in our conceptual framework.

### Provider–Patient Relationship

Patients described CHWs as developing rapport with them by establishing trust and showing humility, compassion, empathy, attentiveness, patience, respect, and altruism. However, one participant did suggest the need for greater supervision to ensure that CHWs speak and listen to patients.

### Trust

… sometimes, you do not want to talk to your husband or your son because they will not understand. Not even the doctor…. The CHW has time to talk to you, and they understand. We are able to talk about problems, and they explain how to manage those problems by understanding us. I trust them to understand my problems (Participant 2)

### Humility and Compassion

So, this [CHW] gave me such a good touch, mainly about diabetes, he was so concerned like a friend, you know, showing me this strength, saying “you have to take care, take care, we think it’s a simple thing and it isn’t” (Participant 8)

### Empathy

Of course [CHWs] can help; they live the day-to-day life I live. Never for a moment did he [CHW] want to play the role of doctor or nurse. He always wanted to collaborate… he understands what it means to be a patient….Of course, it helps, without a doubt having someone who understands what we are going through. (Participant 17)

### Patience and Respect

The CHW said the same thing to me as did the doctor, only in a different way; the CHW is more patient, maybe he’s got more time, the mind is more alert, maybe that’s what it is, it makes the patient have more time to understand things. (Participant 7)

### Attentiveness

The CHW pays more attention to us, she clarifies things; when we have doubts, she takes time, she asks questions, comes and checks everything in my house .. and then she said to me, because of dengue, please watch for the drain, the refrigerator, behind the refrigerator, plants... every little thing … She then comes back to follow up if I have paid attention to her. (Participant 16)

### Altruism

There was one CHW she was great; wow, she was what I needed, she was so selfless she even gave her cell phone, she was not lazy to work. Sometimes it was Sunday, and she came to give me a referral. (Participant 3)

### Provider–Colleague Relationship

Some patients discussed the “politics” inside the local UBS. They reported that physicians, the highest-ranking profession in the FHS, do not see CHWs as equal partners. This is because even though the CHW did his/her job in referring the patient or requesting a home visit by the physician, it was at the physician’s discretion whether or not the service would be provided. This signaled to patients that CHWs were not taken seriously by physicians, undermining their role within the FHS team.

People think CHWs have the power, but they do not. There is a bureaucracy … So the CHW may not have as much strength... they are not taken seriously by physicians. (Participant 18)

The [CHW] was nervous because he had been asking for a doctor to come here for some time, and the doctor never came. Why did not the doctor come? So the CHW does not have much to do for us... the boss [physician] in there, the big one, the head in there, talk to them, make a better team for them to respect one another. This is what is missing a better team that understands everyone on FHS. (Participant 8)

### Provider–Society Relationship

Patients validated that CHWs were able to understand the social determinants of health affecting residents under their care and the prevalence of illnesses. CHWs were described as proactive in mobilizing communities to create change, including teaching residents how to advocate for more and better services.

I think this fact is very important, the CHW does door to door … doing this research, and then they go about educating people not to get the same illness as their neighbors. They alert us. (Participant 4)

We keep our backyard clean, but there is a smell, which is horrible, right? Then I told the CHW, and she said: “Look, we already communicated it to officials but remember you also need to speak to them at city hall, because it is only they who have power to do something, when lots of people complain.” (Participant 6)

Today, they have birds’ poo in the park, which is poisonous; she [CHW] advised us to call the city to ask for cleanup because I have a child who goes to the park, then there is a risk from some animal feces to my child. The CHW also tells us to help clean the park all of us together. (Participant 11)

### Provider–Self Relationship

The key issue in describing the well-being of CHWs was the need for them to engage in self-care. Patients contend that CHWs’ self-care was needed to abate the intense labor involved in walking for several hours every day to make home visits. They also commented on the lack of medical supplies, which creates stress for the CHWs. Several patients acknowledged that the CHW in their catchment area was able to maintain a work-life balance by committing to the stipulated work hours. Patients also underscored the possible low self-esteem felt by CHWs owing to their lack of medical knowledge in resolving patients’ immediate medical needs.

The CHWs work in bad conditions…. walking is very difficult, heat, rain, so many stray dogs on the roads. (Participant 21)

[CHWs] do not take care of themselves… it’s good to follow up on them… to protect them ... from this coming and going every day on foot in rain and heat, you sometimes feel sorry, right? Or, he’s thirsty, and you offer a glass of water, right? Why do they work during lunchtime, then they return again for the afternoon? So, I think they could also have support, right? Look! Like I saw my agent, he went without a hat. When it rained, he didn’t have a raincoat, did he? So that he can have more protection ... so that he doesn’t get sick, right? (Participant 27)

### Countervailing Narratives

A few participants provided, along with the data above, contrasting narratives worth mentioning. These data suggest that some patients wish that CHWs be more attentive and more knowledgeable about medical issues.

There are many CHWs who do not even talk to you much... if she was better supervised, she would pay more attention to what she is doing. (Participant 22)

[CHWs] don’t know everything… This is a big problem for their confidence too... They feel powerless. It is fundamental for our health that we believe what [they] tell us to do. (Participant 1)

## DISCUSSION

As an area for scientific pursuit, NM is short on how community-based providers, such as CHWs, practice the basic tenets of NM.^17^ Our findings narrow this gap by describing how patients perceive CHWs’ use of key NM concepts in four different types of personal and interpersonal relationships—with patients, colleagues, society, and self. With awareness about the limitations of this study (see the section below), this information has the potential to inform strategies for training on key aspects of NM not only for CHWs but also for other community-based health care providers in low-/middle-income countries, such as Brazil.

Of all four NM relationships, patients in this study described the provider–patient one in greater detail. They offered examples of how CHWs were able to show compassion, empathy, attentiveness, patience, respect, and altruism, all of which can build rapport. Patients validated a sense of *trust* between the CHW and patients, especially because CHWs met with patients frequently and for long periods at their homes. They described CHWs as *compassionate—*often placing the needs of their clients ahead of their own. CHWs were described as using *emphatic communication* as they listened to patients, using facial expressions and eye contact along with knowing their names. Good rapport has been described as empowering to patients, which may improve scheduling medical appointments in addition to processing and responding to medical advice.^23^

The composition of FHS teams with varied providers was meant to foster interprofessional collaboration through a synergistic process of combining bio-medical knowledge (physicians, nurses) with experiential knowledge (CHWs).^53^ In Brazil’s unified systems, patients receive medical services in community-based units in the presence of physicians, nurses, and CHWs. This provides patients the capacity to observe power dynamics, grades of professional valuation, trust, interdependence, and other aspects of within-team relationships. Therefore, patients in this study described the provider–colleague relationship as rendering CHWs powerless compared to physicians and nurses. Interprofessional collaboration is an integral part of daily practice in health care.^54^ Efficient interprofessional collaboration has been shown to improve patient outcomes,^55^ provider work satisfaction,^56^ and reduce overall health care costs.^57^ Our study thereby calls for the need for health care providers to recognize the distinct needs of their team members, which may then generate insights to improve team function and dynamics.^58^ For example, in our study, patients advocated for greater communication among CHWs and physicians to ensure that doctors and nurses listen to pressing patient health issues identified by CHWs. When medical staff publicly undermine CHWs, this lack of respect and trust can strongly influence community perceptions of the CHWs’ role and competency.^59^ Patients’ perspectives on the CHW–physician relationship align with other studies involving social providers (e.g., social workers), in which physicians failed to value their colleagues’ skills.^60^ The FHS should encourage and allocate time for team meetings, and for dialogues that may inspire CHWs and medical providers to teach and learn from one another. This may contribute to more agreement about how best to engage, diagnose, treat, and follow-up with patients, as well as how to define and measure patient health behaviors.^19^ Fundamental to this synergistic exchange of knowledge is training that could foster cooperation, mutual respect, clearer communication skills, and division of tasks.^61^

While discussing the provider–society relationship, CHWs appeared to be effective in helping patients address physical, material, and psychosocial factors impacting the health of their communities. NM calls for providers to ask the patient for narratives of their lives and reflect them back to patients as a way to decrease feelings of stigma, marginalization, and exclusion.^62^ CHWS, as this study suggests, systematically assessed the local context^63^ and then mobilized patients to advocate for accessible health services.

The patients in this study provided a small window into the provider–self relationship. They used words, descriptions, and gestures that clearly showed how empathic they felt toward CHWs’ working conditions, such as CHWs walking on foot in the heat and rain and dealing with stray dogs. However, patients in this study also shared feeling a lack of credibility in terms of CHWs’ capacity to perform basic medical tasks (such as taking blood pressure) and functions, for instance, make appointments with specialists. Campaigns with a focus on improving CHWs’ image among residents and colleagues may empower all involved and perhaps showcase CHWs’ altruistic ways of providing valuable services to their communities.

The work of CHWs is deeply rooted in the supportive relationships built with patients, which can be an opportunity for compassion satisfaction (personal accomplishment) and/or professional burnout.^64^ CHWs need training and ongoing supervision to prevent burnout and to ensure that they are routinely performing household visits. Effective supervision is crucial to the success of CHWs in motivating them and enhancing their credibility in the eyes of patients.^59^ Greater flexibility in their work schedules and other supports (e.g., leisure and recreational) from the FHS can help prevent burnout.^65^ While it is burgeoning research based on direct observations of CHWs,^66–68^ we recommend using direct observation of CHW interactions with patients and their colleagues to strengthen the current study’s findings. The CHWs’ personality traits and demographic identities (race, gender, age) should be explored in future research vis-à-vis key interpersonal (trust, repetition, concern, persistence, attentiveness) styles known to advance NM.

### Limitations

We admit that this study lacks a deeper critical lens on how patients may perceive CHWs’ shortcomings and how to overcome them by using NM strategies. This could be attributed to social desirability as patients in this study appeared to be grateful to have their voices heard for a research project. We are cautious that this study includes solely patients’ data (not CHWs’ or physicians’ or any other team members’) on perceptions of the CHW–colleague relationship. Nonetheless, as indicated above, patients are served in the presence of physicians, nurses, and CHWs. This provides patients the capacity to observe power dynamics, grades of professional valuation, trust, and interdependence. Another limitation is the lack of data on how patients perceive the role of government in health care and the specific social ties between CHWs and the communities they serve. This socio-political context would better explain how patients’ perceptions of how the CHW–society relationship might contribute to patients’ perceptions of CHWs’ legitimacy. This important issue needs to be explored in future research.

Furthermore, though diverse in terms of demographic characteristics and a random selection, the study’s sample is limited in scope. However, within the USA, CHWs has become a formal member of the integrated primary health care team; for example, the Patient Protection and Affordable Care Act describes CHWs as members of the health care workforce offering direct patient care and support.^69^ Future research is recommended to evaluate how CHWs apply NM principles in the USA.

## CONCLUSION

This study has expanded our understanding of patients’ perspectives on how community health workers apply narrative medicine techniques. The CHWs can successfully engage in provider–patient and provider–society relationships to engage in personal health behavior changes and advocate for accessible services. Our findings indicate the need for more generous organizational supports to facilitate provider–colleague and provider–self relationships through more significant investment in training focused on cooperation and wellness initiatives in the workplace.
